# Electrophoretic mobility shift assays implicate *XRCC2*:rs3218550C>T as a potential low-penetrant susceptibility allele for sporadic breast cancer

**DOI:** 10.1186/s13104-019-4512-9

**Published:** 2019-08-01

**Authors:** Nirmala D. Sirisena, Nilakshi Samaranayake, Vajira H. W. Dissanayake

**Affiliations:** 1 Human Genetics Unit, Faculty of Medicine, University of Colombo, No. 25 Kynsey Road, Colombo 8, 00800 Sri Lanka; 2 Department of Parasitology, Faculty of Medicine, University of Colombo, Colombo 8, 00800 Sri Lanka

**Keywords:** Alleles, Breast cancer, Genetic variants, Risk, Susceptibility

## Abstract

**Objective:**

A previous study undertaken at our centre to identify common genetic variants associated with sporadic breast cancer in Sri Lankan women showed that the T allele of rs3218550, located in the 3′untranslated region of X-ray repair cross-complementing gene-2 (*XRCC2*), increased breast cancer risk by 1.5-fold. Dual luciferase reporter assays performed in MCF-7 breast cancer cells showed a putative transcriptional repressor effect exerted mainly by the T allele. Electrophoretic mobility shift assays were conducted to further investigate the interaction of this variant with DNA-binding protein, using nuclear protein extracts derived from MCF-7 cells.

**Results:**

An allele-specific differential binding was observed. The T allele resulted in differential DNA–protein complex binding as evidenced by the presence of multiple bands of increased intensity compared to the wild-type C allele. This implies possible alteration in binding of regulatory proteins by the variant allele. These results implicate *XRCC2*:rs3218550C>T as a potential low-penetrant susceptibility allele for sporadic breast cancer. XRCC2 is known to play an essential role in homologous recombination repair of DNA double-strand breaks. It is plausible that this variant may be exerting regulatory effects on *XRCC2* gene expression leading to altered DNA repair capacity. Further functional studies are warranted to validate this finding.

## Introduction

A case–control study was recently undertaken to investigate the association of single nucleotide variants (SNVs) in breast cancer related genes with sporadic breast cancer risk in Sri Lankan women. A biobank consisting of blood samples obtained from postmenopausal women with clinically phenotyped sporadic breast cancer (cases), and postmenopausal women who have never been diagnosed to have any malignancy (controls) was used to examine the association of SNVs with sporadic breast cancer. The results showed that the T allele of rs3218550, located in the 3′untranslated region (3′UTR) of X-ray repair cross-complementing gene-2 (*XRCC2*), increased breast cancer risk by 1.5-fold [1].

Previous studies have reported that SNVs in the DNA repair pathway genes such as *XRCC2* may exert an effect on breast cancer susceptibility by acting as low penetrant alleles [2]. However, the precise functional role of rs3218550 in the XRCC2 protein is unknown as there are no published functional studies implicating a causal relationship between the variant allele and breast carcinogenesis. It is well known that variants in the 3′UTR exert regulatory actions on gene and protein expression through alteration of either transcription factor binding sites, microRNA (miRNA) binding sites or enhancer/promoter effects [3]. 3′UTR SNVs may interfere with messenger RNA (mRNA) stability, mRNA degradation, translational efficiency as well as nuclear export by altering polyadenylation, protein-mRNA and miRNA-mRNA regulatory interactions [4]. Using in silico tools, no highly conserved miRNA binding sites which could be influencing mRNA stability or translation were predicted at this site.

We hypothesized that this SNV may exert transcriptional regulatory effects and its putative functions were further investigated using dual luciferase assays in MCF-7 breast cancer cell lines [5]. The mean relative normalized luciferase activity did not show statistically significant differences between the wild-type and variant alleles. However, a trend was observed which suggested a putative transcriptional repressor effect exerted mainly by the variant T allele of rs3218550 due to its reduced mean luciferase activity compared to the wild-type C allele [5]. We further hypothesized that the observed allele-specific reduction in reporter gene expression could be due to differential binding pattern of transcription regulatory machinery to the 3′UTR DNA sequences at this site. This hypothesis was tested by conducting electrophoretic mobility shift assays (EMSA) to examine the interaction of this variant with DNA-binding protein, using nuclear protein extracts derived from MCF-7 cells.

## Main text

### Methods

In order to investigate whether a single nucleotide variant at the rs3218550 site may alter the protein-DNA interactions, EMSA was performed (Profacgen, USA). The experiments included designing of biotin-labelled, double stranded oligonucleotide probes containing the wild-type and variant allele sequences of the SNV and incubating the probes (in the presence of competitors, i.e. specific probes having the same nucleotide sequence but without biotin labels), with nuclear protein extracts derived from MCF-7 cells. The unlabelled nucleotides were used as specific competitors to eliminate non-specific nucleotide-protein interactions. Protein-DNA complexes were resolved by polyacrylamide gel electrophoresis and detected using a LightShift Chemiluminescent EMSA kit to confirm whether or not any DNA-binding proteins bound to the site.

#### Cell recovery and cell culture

The frozen MCF-7 cells were removed from the liquid nitrogen tank, and quickly put into a 37 °C water bath for 1 min with shaking (to dissolve them quickly). Thereafter, the cell suspension was transferred to a 15 ml sterile centrifuge tube containing 4 ml complete medium. Complete medium consisted of Dulbecco’s modified Eagle’s medium with l-glutamine (1×), 10% heat-treated fetal bovine serum, penicillin (100U/ml), streptomycin (100 pg/ml), 1% non-essential amino acids, and 1 mM sodium pyruvate. The tube was centrifuged at 1000 rpm for 5 min, thereafter the supernatant was discarded and the pellet was re-suspended, and the cells were transferred into a 25 ml culture flask for cultivation.

#### Passaging cells

Cells were grown at a density of 80–90% in the bottom of a 25 ml culture flask, the supernatant was discarded and then 2 ml trypsin was added at 37 °C for 2 min in water bath. The digestion was terminated by adding the culture medium. Thereafter, the cells were centrifuged at 1000 rpm for 5 min. The cells were re-suspended until the number of cells reached 1 × 10^6^. The nuclear protein extraction was performed once the cultured MCF-7 cell count had reached to 1 × 10^6^.

#### Nuclear protein extraction

The sample was washed with pre-cooled phosphate buffered saline and centrifuged at 500×*g* for 3 min, then the supernatant was discarded. Next, 100 μl pre-cooled cytoplasmic extraction reagent I [6] (containing protease inhibitor) was added, vortexed for 15 s, and then placed on ice for 10 min. Thereafter, 5.5 μl pre-cooled cytoplasmic extraction reagent II was added, vortexed for 5 s, and then placed on ice for 1 min. The sample was again vortexed for 5 s, and centrifuged at 16,000×*g* for 10 min. The supernatant was transferred into a pre-cooled centrifuge tube; 40 μl pre-cooled nuclear extraction reagent (including protease inhibitor) added, vortexed for 15 s and then placed on ice for 40 min. Next, the sample was vortexed for 15 s per 10 min and centrifuged at 16,000×*g* for 10 min. The supernatant was transferred into a pre-cooled centrifuge tube, this contained the extracted tissue nuclear protein.

#### Probe preparation

Synthetic biotinylated and non-biotinylated probes were prepared based on the following sequences for the wild-type and variant type alleles of rs3218550:

rs3218550C>T—wild-type allele forward: TGGGCAGTGCTGCAACGAACATACGCGTG. 

rs3218550C>T—wild-type allele reverse: CACGCGTATGTTCGTTGCAGCACTGCCCA.

rs3218550C>T—variant type allele forward: TGGGCAGTGCTGCAATGAACATACGCGTG.

rs3218550C>T—variant type allele reverse: CACGCGTATGTTCATTGCAGCACTGCCCA.

#### Electrophoresis

6% native polyacrylamide gel was prepared and pre-electrophoresis was performed at 4 °C for 60 min with 100 V. The binding reaction solution was prepared, and left at room temperature for 15 min. Biotin-labelled probes were added and left at room temperature for 35 min. Next, 5 μl Loading Buffer was added to each binding reaction, mixed and loaded. Electrophoresis was performed at 4 °C with 100 V, and electrophoresis was stopped when the bromophenol blue indicator reached to two-thirds length of the gel.

#### Transfer of film

The nylon membrane was soaked into 0.5× Tris-borate Ethylenediaminetetraacetic acid (TBE) buffer for 15 min, and the sponge was put, the filter paper, gel, nylon membrane, filter paper and sponge in order according to the sandwich method without bubbles. Transfer was done at 300 mA for 30 min with 0.5× TBE buffer followed by ultraviolet cross-linking.

#### Signal detection

Signal detection refers to the instruction of LightShift^®^ Chemiluminescent EMSA Kit. The blocking buffer and 4× Wash Buffer were pre-heated at 50 °C in water bath, until the particles were completely dissolved. The membrane was blocked with 20 ml blocking buffer for 15 min with shaking on a shaker, thereafter the blocking buffer was discarded. 20 ml blocking buffer which contains 66.7 μl horseradish streptavidin peroxidase was added, and shaken on the shaker for 15 min. The membrane was washed with 20 ml 1× rinse buffer 4 times, each lasting 5 min. The membrane was equilibrated with 30 ml of equilibration buffer for 5 min. The Substrate Working Solution was prepared: 6 ml Luminol/Enhancer Solution with 6 ml Stable Peroxide Solution were mixed and stored in the dark. The membrane was transferred to a new clean dish. The substrate working solution was added for 5 min in the dark. The membrane was put on plastic film without bubbles and wrinkles and the signals were detected.

### Results

**Fig. 1:**
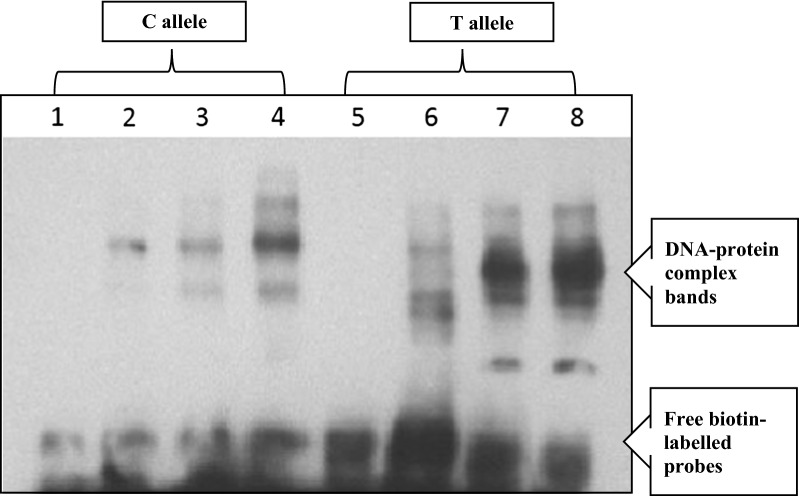
Exposure result of membrane—rs3218550C>T (exposure time: 5 min). Lane 1: probe rs3218550C>T—wild-type allele; Lane 2: probe rs3218550C>T—wild-type allele with specific competition probe (non-biotinylated) and nuclear protein (concentration 1–6 μg/20 μl); Lane 3: probe rs3218550C>T—wild-type allele with nuclear protein (concentration 1—6 μg/20 μl); Lane 4: probe rs3218550C>T—wild-type allele with nuclear protein (concentration 2–12 μg/20 μl); Lane 5: probe rs3218550C>T—variant allele; Lane 6: probe rs3218550C>T—variant allele with specific competition probe (non-biotinylated) and nuclear protein (concentration 1–6 μg/20 μl); Lane 7: probe rs3218550C>T—variant allele with nuclear protein (concentration 1–6 μg/20 μl); Lane 8: probe rs3218550C>T—variant allele with nuclear protein (concentration 2–12 μg/20 μl)

Biotin-labeled, double stranded oligonucleotide probes containing the ancestral and variant allele sequence of rs3218550C>T were incubated (in the presence of competitors, i.e. specific probes having the same nucleotide sequence but without biotin labels) with nuclear protein extracts from MCF-7 cells. The unlabeled nucleotides served as specific competitors to eliminate non-specific nucleotide-protein interactions. Protein-DNA complexes were resolved by polyacrylamide gel electrophoresis and detected using a LightShift Chemiluminescent EMSA kit to confirm whether any DNA-binding proteins bind to the polymorphic site. With the different speed at gel electrophoresis, the interaction between nuclear protein and the DNA probes were analyzed qualitatively. Figure 1 shows the gel shift assay results for rs3218550C>T. Fast migrating unbound probes are found at the bottom of the gel whereas protein–DNA complexes have slower mobility. The results showed that both the wild-type probe and variant-type probe interact with nuclear protein but the data did show allele-specific differential binding. Compared with the wild-type allele C, the variant allele T in rs3218550 showed more binding as indicated by the increased intensity of the bands. In addition, the positions of the bands were also not exactly the same as indicated by multiple bands at different levels which could be due to binding of different proteins at the variant allele site. Competition with the unlabelled probes showed binding specificity corresponding to the sequences at the site.

### Discussion

The results of the EMSA showed an allele-specific difference in DNA–protein binding by rs3218550C>T. Compared with the wild-type C allele, the variant T allele of rs3218550 resulted in differential DNA–protein complex binding, which implies probable alteration of binding of regulatory proteins at this site brought about by the variant allele. An alteration in the protein-DNA interactions between the two alleles implies that the SNV is probably a functional genetic variant exerting some regulatory effects on gene transcription and expression.

*XRCC2* gene encodes the XRCC2 protein, which plays a vital function in the homologous recombination repair (HRR) pathway of double-strand breaks (DSB) in DNA, which aids in maintaining genomic stability [7]. DNA DSB are the most serious form of DNA damage, and non-homologous end joining and HRR are two major repair pathways. It is known that if DNA damage is not repaired correctly, genomic instability can lead to cell cycle arrest, apoptosis, or tumorigenesis [8]. Previous studies have reported that SNVs in the DNA repair pathway genes may act as low penetrant susceptibility alleles for sporadic breast cancer [2]. However, there are no previously published functional studies which report on the exact biological mechanisms of rs3218550 [9].

The XRCC2 protein is one of the five paralogs of RAD51, including RAD51B, RAD51C, RAD51D, XRCC2, and XRCC3. The RAD51 paralogs are all required for efficient DNA DSB repair and depletion of any paralog results in significant decreases in homologous recombination frequency, which may lead to more error-prone repair mechanisms, potentially promoting genomic instability and cancer susceptibility [10, 11]. Hamster cells deficient in XRCC2 have been observed to exhibit defects in RAD51 focus formation, a decrease in HRR induced by DSB, hypersensitivity to radiation and DNA cross-linking agents, increased spontaneous chromosome aberrations, and increased chromosome missegregation. Cells derived from Xrcc2-knockout mice have also been found to exhibit profound genetic instability as a result of HHR deficiency [11]. These studies indicate the critical roles of XRCC2 in HRR [12].

Based on the results of this study, it is proposed that the variant T allele of rs3218550 may affect DNA repair capacity by regulating *XRCC2* gene expression and thus, influence an individual’s susceptibility to sporadic breast cancer. It is also possible that this SNV may be exerting its effect by acting singly or in synergy with other unknown functional variants which are in linkage disequilibrium with it. These results implicate *XRCC2*:rs3218550C>T as a potential low-penetrant susceptibility allele for sporadic breast cancer. It is possible that the variant T allele may influence an individual’s susceptibility to sporadic breast cancer through altered HRR capacity. However, there might be other biological pathways involved, and further functional studies are warranted to elucidate the precise functional effects on the XRCC2 protein and to establish a definite causal effect.

## Limitations

- As EMSA is considered a qualitative test, it is not possible to comment on the binding affinity. To measure this binding event quantitatively, an advanced technique such as surface plasmon resonance (SPR) would need to be performed. SPR, is the gold standard for binding affinity and kinetics measurements [13].
- Supershift analysis using antibodies was not done as the identities of the DNA binding proteins were unknown.
- Identifying the binding proteins by mass spectrometry and testing for differential binding in vivo were not done.
- Conducting further experiments to determine the effect of this SNV on RNA stability or translation were not done.

## Data Availability

The datasets used and/or analyzed during the current study are available from the corresponding author on reasonable request.

## Abbreviations

3′UTR: 3′untranslated region
DSB: double-strand breaks
EMSA: electrophoretic mobility shift assay
HRR: homologous recombination repair
mRNA: messenger RNA
miRNA: microRNA
SNVs: single nucleotide variants
SPR: surface plasmon resonance
TBE: Tris-borate Ethylenediaminetetraacetic acid
*XRCC2*: X-ray repair cross-complementing gene-2
